# The relationship between professional quality of life and work environment among ICU nurses in Chinese: a cross-sectional study

**DOI:** 10.3389/fpubh.2023.1104853

**Published:** 2023-05-04

**Authors:** Weiwei Ni, Ming Xia, Mengjuan Jing, Shichao Zhu, Liming Li

**Affiliations:** Intensive Care Unit, Henan Provincial People's Hospital, People's Hospital of Zhengzhou University, Zhengzhou, China

**Keywords:** professional quality of life, nursing working environment, intensive care unit, nurse, China

## Abstract

**Objective:**

To explore the relationship between the professional quality of life and work environment among intensive care unit nurses, and identify the influencing factors of intensive care unit nurses' professional quality of life.

**Methods:**

This study design is cross-sectional and correlational descriptive. Four hundred fourteen intensive care unit nurses from Central China were recruited. Data were collected from three questionnaires of self-designed demographic questions, the professional quality of life scale and the nursing work environment scale. Descriptive statistics, Pearson's correlation, bivariate analysis and multiple linear regression were used to analyze the data.

**Results:**

A total of 414 questionnaires was collected, for an effective recovery rate of 98.57%. The original scores of the three sub-scales of professional quality of life were 33.58 ± 6.43, 31.83 ± 5.94, and 32.55 ± 5.74. Compassion satisfaction was positively correlated with the nursing working environment (*p* < 0.05), job burnout, and secondary trauma were negatively correlated with nursing work in environment (*p* < 0.05). Multiple linear regression analysis results show that, the nursing working environment entered into the influential factor model of professional quality of life scale (*p* < 0.001). The nursing working environment independently explained 26.9% of the changes in compassion satisfaction, 27.1% of the changes in job burnout, and 27.5% of the changes in secondary trauma. The nursing work environment is an important factor affecting the professional quality of life.

**Conclusion:**

The better the nursing working environment, the higher the professional quality of life of intensive care unit nurses. Decision makers and managers can focus on improving the working environment of nurses, which may be a new perspective for managers to improve the professional quality of life of nurses and stabilize the nursing team.

## 1. Introduction

According to the “Healthy China 2030 plan,” nurses play an important role in meeting the diverse and differentiated health needs of the people. As of 2020, the number of registered nurses in China is 4.709 million, accounting for about 1/4 of the global nursing staff (1). The number of nurses per 1,000 population in China is less than three, and they shoulder the responsibility of protecting the health of 1.4 billion people. Compared with nurses in developed countries in Europe and the United States, Nurses in China are facing more severe tasks (2). ICU as a place to treat critically ill patients, ICU nurses have become the main force in treating critically ill patients and responding to public health emergencies. It is necessary to allocate adequate nursing human resources to improve the treatment effect and nursing quality of critically ill patients. The continuous development of critical care medicine has put forward higher requirements for ICU nurses' professional quality and professional ability (3, 4). Under the background of ICU human resource shortage, how to improve the nursing working environment and improve the professional quality of life of ICU nurses is particularly important.

Professional quality of life (ProQoL) refers to the quality of life a person feels in the process of helping others and its impact on mental health. It is a new way to evaluate nurses' occupational health in multiple dimensions (5). For intensive care unit (ICU) nurses, ProQoL is composed of compassion satisfaction and compassion fatigue (6). Compassion satisfaction refers to the self-satisfaction that ICU nurses obtain in the process of providing nursing care to patients and is a positive factor (7). Compassion fatigue is composed of job burnout and secondary trauma. Job burnout is a syndrome of mental, physical and emotional exhaustion as a result of a state of chronic work stress (8). Secondary trauma is the fear and helplessness caused by indirect exposure to traumatic stress. Compassion fatigue is a negative factor (9, 10). Research shows that the nurse ProQoL is at a low to medium level, which affects nurses' work enthusiasm and nursing quality (4, 11). Paying attention to and improving nurse ProQoL is conducive to protecting nurses' rights and interests, promoting nurses' physical and mental health, reducing the incidence of job burnout, enhancing professional happiness, and thus promoting nursing team construction and improving nursing quality. Recent studies on nurse ProQoL mainly focus on emergency department, oncology department, obstetrics department, intensive care unit, psychiatry department and pediatrics department. The ProQoL level of nurses in oncology department was low, and the incidence of moderate and severe job burnout and secondary trauma were 62.76 and 66.84%, respectively (5). The ProQoL of psychiatric nurses was on the medium level, with the lowest score in the secondary trauma dimension (12). The overall ProQoL of Chinese nurses was at a low to medium level. The average score of ProQoL items of nurses in the intensive care unit was (3.35 ± 0.44), which was at the lower medium level, and the score of compassion satisfaction scale was the lowest (13). Factors affecting nurse ProQoL mainly include working environment, management style, leadership style, salary and benefits, promotion opportunities, occupational safety, workload, scheduling mode, team atmosphere, etc. (5, 11). Insufficient manpower, heavy workload, frequent night shifts and long weekly working hours will cause physical and mental exhaustion of nurses and reduce their ProQoL (5). The public attitude that recognizes the value of nurses and respects their labor can improve nurse ProQoL (14). In general, there are differences in results among different countries, regions, hospital grades, departments, seniority and gender. Nurses in emergency, intensive care, oncology, psychiatry and other intensive work departments have a low ProQoL level, which should be paid attention to by nursing managers.

The nursing work environment (NWE) is the physical, chemical, biological, organizational, social, and cultural factors that surround a worker (15). According to the American Association of Critical-Care Nurses (AACN), “a healthy work environment (HWE) is imperative to ensure patient safety, enhance staff satisfaction and retention, and maintain an organization's financial viability” (16). The ICU is a recognized high-stress work environment. Nurses have a heavy workload and require a high level of nursing skills and abilities. The recipients of care are critically ill patients with life-threatening conditions, and nurses are always exposed to secondary trauma at work as a result of resuscitation, pain, major surgery, and the death of patients and the grief of their families (17). A large number of studies (18–21) confirmed that compared with nurses who work in a less stressful environment, nurses who work in a more stressful environment, such as the ICU, are more likely to experience mental and physical exhaustion, which affects their ProQoL and care giving capacity. In 2005, the AACN issued six standards for the establishment and maintenance of a HWE and required health care leaders to stringently assess environmental conditions and provide clear and measurable methods for improving working conditions (16). Monroe et al. (6) studied the relationship between the six HWE standards in the ICU and the ProQoL of nurses. The results showed that the working environment affected the ProQoL of nurses, and genuine cooperation, effective decision-making, and leadership are the indicators with the strongest influence on ProQoL.

In summary, job burnout and turnover of nurses are important variables in human resource management in nursing institutions. ICU nurses work under high pressure and maintain satisfaction with their responsibilities, which is largely influenced by various environmental factors. NWE is one of the most important external factors that affect nurse job burnout. A supportive work environment can reduce nurse job burnout and is an important predictor of job satisfaction and active nursing. NWE is closely related to the health and quality of life of nurses, and indirectly affects patient satisfaction and survival rate. However, there is relatively little research on the relationship between the ProQoL and NWE of ICU nurses in China, and there is no research on the relationship between NWE and compassion satisfaction. This study also considers multiple factors such as staffing, leadership and management, and career development in the work environment. The practical strategy for establishing a good ProQoL should be based on the analysis of the relationship between ProQoL and NWE. Therefore, the main objectives of this study were: (a) to explore the level of ProQoL and NWE of ICU nurses; (b) To explore the relationship between ProQoL of ICU nurses and NWE; (c) The influence of social demographic factors and NWE factors on ICU nurses' ProQoL. Further optimizing the working environment of ICU nurses may be a new perspective for managers to improve the ProQoL of nurses and stabilize the nursing team.

## 2. Methods

### 2.1. Study design

This study design is cross-sectional and correlational descriptive.

### 2.2. Participants

The convenience sampling method was used to select ICU nurses from 12 hospitals in Henan Province from February to March 2021 as the study subjects. The inclusion criteria were as follows: (a) aged 18 to 60 years old; (b) ongoing work in ICU nursing for ≥ 6 months; and (c) no cognitive impairment or language communication impairment; (d) Subjects volunteered to participate in this study. According to the principle that the sample size of a study should be 5–10 times the number of dependent variables (22), this study required at least 150–300 participants. The estimated sample size was increased to account for sample loss and missing data. Based on the actual situation, 420 participants were eventually included in the survey. The study was reviewed and approved by the Ethics Committee of Henan Provincial People's Hospital (2021-209). Participants provided informed consent to participate in this study.

### 2.3. Measurements

#### 2.3.1. Demographic and work-related characteristics

The following demographic information was collected from each participant: gender, age, education, marital status, years of work, job title, nature of employment, monthly income, hospital level, and nature of the work unit.

#### 2.3.2. Professional quality of life scale

The Chinese version of the ProQoL Scale developed by Shen et al. (23) was used in the study. It includes compassion satisfaction (10 items), job burnout (10 items), and secondary trauma (10 items) sub-scales, with a total of 30 items. A 5-point Likert scoring system was adopted, and ratings from 1 to 5 represent “never,” “rarely,” “sometimes,” “often,” and “always,” respectively. Items 1, 4, 15, 17, and 29 were reverse scored, and the remaining items were forward scored. Each dimension is scored separately. After calculating the original score and converting it into the Z score, calculate the standard score according to the formula “standard score (T) = 10Z + 50.” Standard score < 43 was divided into low group, 43~57 was divided into medium level, >57 is divided into high group. Cronbach's α of the three sub-scales of ProQoL are 0.82, 0.73, and 0.76. In this study, the Cronbach's α coefficients of the three sub-scales were 0.88, 0.86, and 0.85.

#### 2.3.3. Nursing work environment scale

The NWE scale was measured using the Work Environment Scale of the Nursing Work Index developed by Shao et al. (24). The scale has seven sub-scales (career development, leadership and management, doctor-nurse relationship, Recognition of atmosphere, professional autonomy, basic guarantee, and sufficient manpower) and includes 26 items. A 6-point Likert scale with scores ranging from very “dissatisfied” to “very satisfied” was used to score for each item. All items were forward scored, and the scores of the 26 items were added together. The higher the score was, the better the working environment. Cronbach's α of NWE total scale were 0.946, and Cronbach's α of seven sub-scales were 0.799~0.896. In this study, The Cronbach's α was 0.98 for this scale and was 0.92–0.96 for the sub-scales.

### 2.4. Data analysis

The data in the Wen juanxing platform (https://www.wjx.cn/) were exported as excel spreadsheets for preliminary data collation. The collated data were imported into SPSS 26.0 statistical software for statistical analysis. Count data are expressed as frequencies and percentages, and measurement data are expressed as means ± standard deviations. Analysis of variance was used to compare the ProQoL scores of ICU nurses with different demographic characteristics. Pearson correlation analysis was used to compare the relationship between the ProQoL of ICU nurses and the NWE. Multivariate linear regression analysis was used to study the impact of the NWE on ProQoL.

## 3. Results

### 3.1. Sample characteristics

A total of 414 nurses returned questionnaires from the 12 hospitals. There were 65 males and 349 females, accounting for 15.7 and 84.3% of the sample, respectively. The age group was mainly between 25 and 30 years old, accounting for 49.8%; The average length of ICU nursing work was 6.06 ± 3.5 years. Demographics are shown in Table 1.

**Table 1:** Socio-demographic characteristics and descriptive variables for the survey (*n* = 414).

**Category**		**Number of people**	**Percentage**
Age, years	< 25	45	10.9
	25~30	206	49.8
	31~35	133	32.1
	36~40	24	5.8
	>41	6	1.4
Sex	Male	65	15.7
	Female	349	84.3
Education	Associate degree	27	6.5
	Bachelor	371	89.6
	Master	14	3.4
	PhD	1	0.5
Title	Nurse	44	10.6
	Nurse practitioner	194	46.9
	Nurse supervisor	171	41.3
	Co-chief superintendent nurse	5	1.2
Marital status	Unmarried	170	41.1
	Married	244	58.9
Nature of employment	Contractor	176	42.5
	Personnel agency	229	55.3
	Staffing at public institution	9	2.2
Monthly income (yuan/month)	< 4,000	1	0.2
	4,001~8,000	84	20.3
	8,001~12,000	197	47.6
	12,001~16,000	89	21.5
	>16,000	43	21.5
Hospital level	Primary hospital	10	21.5
	Secondary hospital	58	14.0
	Tertiary hospital	346	83.6
Type of hospital	General hospital	354	85.5
	Specialty hospital	60	14.5
		X ± s (Original score)	
The average length of ICU nursing work (years)		6.06 ± 3.5	
Professional quality of life	Compassion satisfaction	33.58 ± 6.43	
	Job burnout	31.83 ± 5.94	
	Secondary trauma	32.55 ± 5.74	
The nursing work environment	Career development	24.92 ± 4.57	
	Leadership and management	19.20 ± 3.91	
	Doctor-nurse relationship	18.38 ± 3.94	
	Recognition of atmosphere	14.44 ± 2.61	
	Professional autonomy	19.47 ± 3.21	
	Basic guarantee	13.34 ± 3.28	
	Sufficient manpower	13.27 ± 3.13	
	Summary	123.03 ± 21.66	

### 3.2. Analysis of the correlation between ICU nurses' professional quality of life and the nursing work environment

The Pearson correlation analysis results of the three sub-scales of the ProQoL scale, the total score of the NWE scale and the seven sub-scales are shown in Table 2. There was a significant positive correlation between compassion satisfaction and the total score of the NWE scale (r = 0.498, *p* < 0.001). There was a significant negative correlation between job burnout and the total score of the NWE scale (r = −0.473, *p* < 0.001). There was a significant negative correlation between secondary trauma and the total score of the NWE scale (r = −0.418, *p* < 0.001).

**Table 2:** Correlation analysis of ICU nurses' professional quality of life and the nursing work environment (*r* value, *n* = 414).

**Sub-scales of ProQOL**	**Total score of NWE scale**	**Career development**	**Leadership and management**	**Doctor-nurse relationship**	**Environment of recognition**	**Professional autonomy**	**Basic guarantees**	**Sufficient manpower**
Compassion Satisfaction	0.498^*^	0.395^*^	0.385^*^	0.427^*^	0.462^*^	0.452^*^	0.456^*^	0.505^*^
Job burnout	−0.473^*^	−0.376^*^	−0.369^*^	−0.405^*^	−0.459^*^	−0.437^*^	−0.423^*^	−0.466^*^
Secondary trauma	−0.418^*^	−0.329^*^	−0.322^*^	−0.360^*^	−0.388^*^	−0.392^*^	−0.374^*^	−0.425^*^

ProQOL, Professional Quality of Life; NWE, Nursing Work Environment Scale.

* *p* < 0.001.

### 3.3. Analysis of the factors influencing ICU nurses' professional quality of life

The three sub-scales of the ProQoL scale were taken as the dependent variables to conduct multiple linear regression analysis. First, variables with statistical significance (*p* < 0.05) in the bivariate analysis were included as control variables. The assignment method is shown in Table 3. Then, NWE variables with statistical significance (*p* < 0.05) were included in the correlation analysis. After excluding the mixed interference of age, education, professional title, marital status, employment nature, monthly income (yuan/month), hospital grade, etc. NWE entered into the influential factor model of compassion satisfaction scale (*F* = 17.30, *p* < 0.001). It could independently explain 26.9% of the variation of compassion satisfaction, and the score of career development had a significant positive effect on the compassion satisfaction (β = 0.151 > 0, *p* < 0.05). The score of leadership and management had a significant positive effect on the compassion satisfaction (β = 0.075 > 0, *p* < 0.05). The score of the dimension of professional autonomy significantly positively affected the compassion satisfaction (β = 0.135 > 0, *p* < 0.05). The score of sufficient manpower had a significant positive effect on compassion satisfaction (β = 0.222 > 0, *p* < 0.001). The NWE entered into the influencing factor model of job burnout scale (F = 19.61, *p* < 0.001), which could independently explain the 27.1% variation of job burnout. Among which, the score of career development dimension had a significant negative effect on the job burnout (β = −0.157 < 0, *p* < 0.05). The score of sufficient manpower had a significant negative effect on job burnout (β = −0.222 < 0, *p* < 0.001). The NWE was included in the secondary trauma scale model (F = 18.94, *p* < 0.001), which could independently explain 27.5% of the variation in secondary trauma. Among which, the score of career development dimension had a significant positive effect on the satisfaction of secondary trauma (β = 0.162 > 0, *p* < 0.05). The score of leadership and management had a significant negative effect on secondary trauma (β = −0.085 < 0, *p* < 0.05). The score of the dimension of nursing relationship had a significant positive effect on secondary trauma (β = 0.024 > 0, *p* < 0.001). The score of sufficient manpower had a significant negative effect on secondary trauma (β = −0.232 < 0, *p* < 0.001) shown in Table 4.

**Table 3:** Assignment method for independent variables.

**Control variable**	**Assignment**
Age	<25 (X1 = 1, X2 = 0, X3 = 0, X4 = 0, X5 = 0); 25~30 (X1 = 0, X2 = 1, X3 = 0, X4 = 0, X5 = 0); 31~35 (X1 = 0, X2 = 0, X3 = 1, X4 = 0, X5 = 0); 36~40 (X1 = 0, X2 = 1, X3 = 0, X4 = 1, X5 = 0); >41 (X1 = 0, X2 = 0, X3 = 0, X4 = 0, X5 = 0)
Education	Associate degree (X1 = 1, X2 = 0, X3 = 0, X4 = 0); Bachelor (X1 = 0, X2 = 1, X3 = 0, X4 = 0); Master (X1 = 0, X2 = 0, X3 = 1, X4 = 0); PhD (X1 = 0, X2 = 0, X3 = 0, X4 = 0)
Title	Nurse (X1 = 1, X2 = 0, X3 = 0, X4 = 0); Nurse practitioner (X1 = 0, X2 = 1, X3 = 0, X4 = 0); Nurse supervisor (X1 = 0, X2 = 0, X3 = 1, X4 = 0); Co-chief superintendent nurse (X1 = 0, X2 = 0, X3 = 0, X4 = 0)
Marital status	Unmarried (X1 = 1, X2 = 0), Married (X1 = 0, X2 = 0)
Nature of employment	Contractor (X1 = 1, X2 = 0, X3 = 0), Personnel agency (X1 = 0, X2 = 1, X3 = 0), Staffing at public institution (X1 = 0, X2 = 0, X3 = 0)
Monthly income (yuan/month)	<4,000 (X1 = 1, X2 = 0, X3 = 0, X4 = 0, X5 = 0); 4,001~8,000 (X1 = 0, X2 = 1, X3 = 0, X4 = 0, X5 = 0); 8,001~12,000(X1 = 0, X2 = 0, X3 = 1, X4 = 0, X5 = 0); 12,001~16,000(X1 = 0, X2 = 1, X3 = 0, X4 = 1, X5 = 0); >16,000(X1 = 0, X2 = 0, X3 = 0, X4 = 0, X5 = 0)
Hospital level	Primary hospital (X1 = 1, X2 = 0, X3 = 0), Secondary hospital (X1 = 0, X2 = 1, X3 = 0), Tertiary hospital (X1 = 0, X2 = 0, X3 = 0)
The nursing work environment	Original value entry

**Table 4:** Multivariate regression analysis of factors influencing ICU nurses' professional quality of life (*n* = 414).

**Model**		**Unstandardized coefficient**		**Normalization coefficient**	***t***	***p***	**VIF**	**Adjusted *R*^2^**	***F***	***p***
		β	**Standard error**	**Beta**						
Dependent variable (compassion satisfaction)								0.269	17.30	0.001
Independent variable	Career development	0.151	0.051	0.085	1.102	0.016^*^	3.208			
	Leadership and management	0.075	0.053	−0.129	−1.518	0.033^*^	3.967			
	Doctor-nurse relationship	0.024	0.052	0.043	0.466	0.656	3.592			
	Environment of recognition	0.069	0.061	0.065	0.727	0.461	5.122			
	Professional autonomy	0.135	0.067	0.199	2.047	0.041^*^	4.589			
	Basic guarantees	−0.052	0.053	−0.089	−0.932	0.332	5.589			
	Sufficient manpower	0.222	0.052	0.402	4.258	0.000^**^	4.223			
Dependent variable (job burnout)								0.271	19.61	0.001
Independent variable	Career development	−0.157	0.054	−0.090	1.114	0.013^*^	3.310			
	Leadership and management	0.267	0.051	0.118	1.318	0.057	3.956			
	Doctor-nurse relationship	0.021	0.072	0.079	2.461	0.056	3.792			
	Environment of recognition	0.049	0.068	0.075	0.725	0.469	5.122			
	Professional autonomy	0.138	0.067	0.199	2.042	0.052	4.579			
	Basic guarantees	0.052	0.057	−0.099	−0.912	0.362	5.689			
	Sufficient manpower	−0.222	0.052	−0.402	4.257	0.000^**^	4.320			
Dependent variable (secondary trauma)								0.275	18.94	0.001
Independent variable	Career development	0.162	0.047	0.086	1.121	0.017^*^	3.202			
	Leadership and management	−0.085	0.053	−0.137	−1.418	0.041^*^	3.457			
	Doctor-nurse relationship	0.024	0.052	0.041	0.460	0.046^*^	3.763			
	Environment of recognition	0.038	0.063	0.078	0.726	0.469	5.001			
	Professional autonomy	0.141	0.057	0.123	2.052	0.051	4.342			
	Basic guarantees	−0.062	0.051	−0.103	−0.972	0.362	5.235			
	Sufficient manpower	−0.232	0.057	−0.419	4.242	0.000^*^	4.814			

* *p* < 0.05,

** *p* < 0.01.

## 4. Discussion

This study was a cross-sectional and correlational descriptive study in China that involved ICU nurses from 12 hospitals in different geographic regions. The results showed that age, educational background, professional title, marital status, employment nature, monthly income (yuan / month) and hospital level had an impact on ICU nurses' ProQoL, but the conclusions were different from previous studies (6, 11–13). Lebni et al. (11) found that female nurses had higher ProQoL than male nurses, and married nurses had higher ProQoL. Sukut et al. (13) found ProQoL of nurses with different ages and working years, and the difference was not statistically significant. However, some studies (6, 12, 23) found that age was a predictor of ProQoL of nurses. The reason for this result may be influenced by different country, region and hospital culture.

Consistent with most other research on similar topics (6, 14, 20, 21), ICU nurses have higher secondary traumatic stress. This finding suggests that working in the ICU is demanding and nurses are prone to stress. Correlation analysis results of this study show that there was significant correlation between the score on the ProQoL scale and the score of the NWE scale and scores of all sub-scales. There was a significant positive correlation between the score of compassion satisfaction sub-scale in ProQoL and the score of seven sub-scales of NWE scale (career development, leadership and management, nursing relationship, recognition environment, professional autonomy, basic security, sufficient manpower). On the contrary, job burnout and secondary trauma scores were negatively correlated with the seven sub-scales of the NWE scale. It is further explained that ProQoL in ICU nurses is associated with a good work environment. This is consistent with Monroe et al. (6). These results suggest that the better the ICU work environment, the higher the nurses' compassion satisfaction, and the lower the incidence of burnout and secondary trauma. Therefore, nursing managers should focus on improving the NWE to improve the satisfaction of ICU nurses' compassion satisfaction and reduce their job burnout and secondary trauma.

The Institute of Medicine summarizes the development of the theory and practice of NWE, and believes that NWE is the product of hospital management practice, nursing staff deployment, job design and organizational culture, and is an important premise to ensure the quality of patient care (6, 12). The results of multiple linear regression analysis in this study showed that NWE was an important predictor of ProQoL after excluding confounding factors such as age, education, professional title, marital status, employment nature, monthly income (yuan/month) and hospital level.

The findings of this study, career development, leadership and management, professional autonomy, and manpower adequacy have significant effects on ICU nurse ProQoL, which is consistent with a number of previous studies (10, 16, 20, 21). Interestingly, this study found that career development had an effect on compassion satisfaction, job burnout and secondary trauma, which is similar to Monroe et al. (6). This shows that we should pay attention to the improvement of nurses' professional level and comprehensive ability, build a platform for professional development such as further study, training and innovation, and improve nurse ProQoL by providing a supportive environment. Therefore, it is suggested that hospital managers should jointly carry out multi-departments such as Human Resources Department, Nursing Department and Operation Improvement Department, and apply standardized career development planning tools to nurse career development planning projects, which can help meet the needs of new nurses and provide new career development opportunities for senior nurses.

In addition, this study found that sufficient manpower also had an impact on compassion satisfaction, job burnout, and secondary trauma, which was consistent with the study by Tucker et al. (25). Insufficient manpower, heavy workload, high night shift frequency and long weekly working hours will cause physical and mental exhaustion of nurses and reduce their ProQoL (5, 18, 20). The results showed that insufficient manpower and unreasonable staffing increased the incidence of complications and decreased the ProQoL of nurses. Therefore, it is particularly important for ProQoL to strengthen the construction of the nursing team, especially to supplement the human resources of ICU nurses.

Leadership and management also influence compassion satisfaction and secondary trauma in this study. This is similar to the study of Buckley et al. (26), which adopted hierarchical management, service-oriented leadership, magnetic management and other modes to improve the results on the nurse ProQoL. At present, studies have formed a nursing management evaluation system based on magnetic certification evaluation model (8, 17, 27). In the future, China can learn from foreign mature models, build localized certification standards suitable for Chinese cultural background, and strengthen the management mode of hospitals.

Job autonomy refers to the ability of nurses to complete work independently, arrange work methods and procedures independently, and make independent decisions and judgments during work (7). Abbasi et al. (28) investigated 750 nurses in emergency department and intensive care unit, and found that nurses with scientific working methods, reasonable time allocation, strong learning ability, and overall control over work had higher ProQoL level. The reason may be that nurses with strong job autonomy are more likely to obtain leadership, organizational support and patient trust, and stronger job satisfaction and professional identity can have a positive impact on the nurse ProQoL (7, 15).

These results indicate that the influence of nursing work environments of ICU nurses ProQoL is multifaceted. In view of this, the future work goal of nursing managers is to strengthen the construction of the ICU nurse team, pay attention to person-post matching, allocate corresponding responsibilities, rights and benefits in place, and enhance nurses' work autonomy and sense of empowerment. Make arrangements for nurses of different ages and seniority, and take into account nurses with special needs; Implement a performance-based distribution system of high performance and high pay; Implement dynamic flexible scheduling according to the workload, strictly define the shift time; Improving the safety of the NWE; Improve the hospital management evaluation system and other ways to improve ICU nurse ProQoL.

## 5. Limitations

An important limitation of this study is that it is a cross-sectional study and cannot assess the temporal order of observations or determine causal relationships. Further studies using longitudinal data are needed to confirm the causal relationship suggested by our findings.

## 6. Implication for nursing management

This study conducted a large sample survey of ICU nurses' ProQoL and NWE. The study found that there were differences in the ProQoL of ICU nurses with different demographic characteristics, that there was a significant correlation between the NWE and the ICU nurses' ProQoL, and that the NWE is the most important predictor of ICU nurses' ProQoL. This suggests that ICU nursing managers should pay attention to the NWE, stringently assess environmental conditions, and provide clear and measurable methods for improving the NWE to maximize ICU nurses' ProQoL.

## 7. Conclusion

The results of this study showed that the ProQoL of ICU nurses was at a moderate level. Factors that affect ICU nurses' ProQoL include career development, leadership and management, professional autonomy, and sufficient manpower. The better the NWE, the higher the ProQoL of ICU nurses, nurse managers should optimize the NWE, improve the ProQoL of ICU nurses, improve the quality of ICU nursing. Further longitudinal or experimental designs measure additional potential confounding variables to enhance the validity of the understanding of ICU nurses' ProQoL.

## Data availability statement

The original contributions presented in the study are included in the article/supplementary material, further inquiries can be directed to the corresponding authors.

## Ethics statement

This study was approved by Ethics Committee of Henan Provincial People's Hospital (2021-209). Written informed consent for participation was not required for this study in accordance with the national legislation and the institutional requirements.

## Author contributions

LL and WN conceived and designed the experiments, drafted the manuscript, and approved the final version. WN, MX, and SZ performed the experiments, analyzed, and interpreted the data. MJ and MX contributed reagents/materials/analysis tools and revised the manuscript. All authors have approved the final version of the manuscript.
